# Kinetic study of batch and fed-batch enzymatic saccharification of pretreated substrate and subsequent fermentation to ethanol

**DOI:** 10.1186/1754-6834-5-16

**Published:** 2012-03-20

**Authors:** Rishi Gupta, Sanjay Kumar, James Gomes, Ramesh Chander Kuhad

**Affiliations:** 1 Lignocellulose Biotechnology Laboratory, Department of Microbiology, University of Delhi South Campus, New Delhi 110021, India; 2 Kusuma School of Biological Sciences, Indian Institute of Technology Delhi, New Delhi 110016, India

**Keywords:** Enzymatic hydrolysis, Fed-batch, Kinetic model, Fermentation, Delignified substrate, Bioethanol

## Abstract

**Background:**

Enzymatic hydrolysis, the rate limiting step in the process development for biofuel, is always hampered by its low sugar concentration. High solid enzymatic saccharification could solve this problem but has several other drawbacks such as low rate of reaction. In the present study we have attempted to enhance the concentration of sugars in enzymatic hydrolysate of delignified *Prosopis juliflora*, using a fed-batch enzymatic hydrolysis approach.

**Results:**

The enzymatic hydrolysis was carried out at elevated solid loading up to 20% (w/v) and a comparison kinetics of batch and fed-batch enzymatic hydrolysis was carried out using kinetic regimes. Under batch mode, the actual sugar concentration values at 20% initial substrate consistency were found deviated from the predicted values and the maximum sugar concentration obtained was 80.78 g/L. Fed-batch strategy was implemented to enhance the final sugar concentration to 127 g/L. The batch and fed-batch enzymatic hydrolysates were fermented with *Saccharomyces cerevisiae *and ethanol production of 34.78 g/L and 52.83 g/L, respectively, were achieved. Furthermore, model simulations showed that higher insoluble solids in the feed resulted in both smaller reactor volume and shorter residence time.

**Conclusion:**

Fed-batch enzymatic hydrolysis is an efficient procedure for enhancing the sugar concentration in the hydrolysate. Restricting the process to suitable kinetic regimes could result in higher conversion rates.

## Background

Production of cellulosic ethanol from lignocellulosic biomass represents a potential alternative to the petroleum fuel due to its renewable nature and sustainable availability. Currently, the major strategy used for cellulosic ethanol production includes three main steps i.e., biomass pretreatment, enzymatic hydrolysis and ethanol fermentation [1,2]. The enzymatic hydrolysis contributes significantly to the cost of cellulosic ethanol and from the process economics perspective, the improvement in the enzymatic hydrolysis step is a prerequisite [3,4]. The main obstacles for enzymatic hydrolysis are low rate of reaction, high cost of enzyme, low product concentration and lack of understanding of cellulase kinetics on lignocellulosic substrates [5,6]. One way to overcome this problem is to operate the enzymatic hydrolysis using high insoluble solid consistency [7-9]. However, the saccharification reaction at high insoluble solid consistency will have to encounter the problems of increased viscosity, higher energy requirement for mixing, shear inactivation of cellulases, and poor heat transfer due to rheological properties of dense fibrous suspension [9,10].

Interestingly in fed-batch enzymatic hydrolysis such problems could be avoided by adding the substrate and/or enzymes gradually to maintain the low level of viscosity [11]. The fed-batch enzymatic saccharification process has several other economic advantages over conventional batch process such as lower capital cost due to reduced volume, lower operating costs and lower down-stream processing cost due to higher product concentration [6,7]. There are several reports on fed-batch enzymatic saccharification which mainly deal with the development of appropriate kinetic models for mechanistic description of the phenomena [9,12,13]. However, the reports on process operation, optimization and control for fed-batch enzymatic saccharification are scarce [14]. Till date, the strategies used for fed-batch enzymatic saccharification are categorized into three main groups i.e., (i) to recycle enzyme; (ii) fed-batch SSF to mitigate inhibitory effect and (iii) fed-batch saccharification to increase the cumulative substrate in a reactor [7]. Here, the present study falls within the third category and our main emphasis was to enhance the total solid content and sugar concentration, which eventually resulted in higher ethanol production.

The experimental data on cellulose hydrolysis by cellulases point to various bottlenecks that decrease the rate of conversion. Mathematical modeling of the enzymatic hydrolysis process is an important tool for analyzing these bottlenecks [5]. Use of mathematical modeling can lead to several advantages viz. the effect of feeding profiles on sugar conversion can be evaluated apriori, kinetics of the hydrolysis process can be studied and process simulations can be made to understand the kinetic regimes. Recently, Hodge and colleagues [7] have used model based fed-batch approach to develop a feeding profile for the fed-batch enzymatic saccharification, while, Morales-Rodriguez and coworkers [14] used a modeling approach to reduce the amount of enzyme during the fed-batch enzymatic saccharification.

The present study deals with the development of the feeding profile and a mathematical model for the understanding of the enzymatic saccharification kinetics in a stirred tank reactor (STR). Moreover, the hydrolysates obtained after batch and fed-batch enzymatic hydrolysis has subsequently been fermented to ethanol, and an overall comparison between batch and fed-batch process has been presented.

## Results

### Kinetics of batch and fed-batch enzymatic hydrolysis

**Figure 1:**
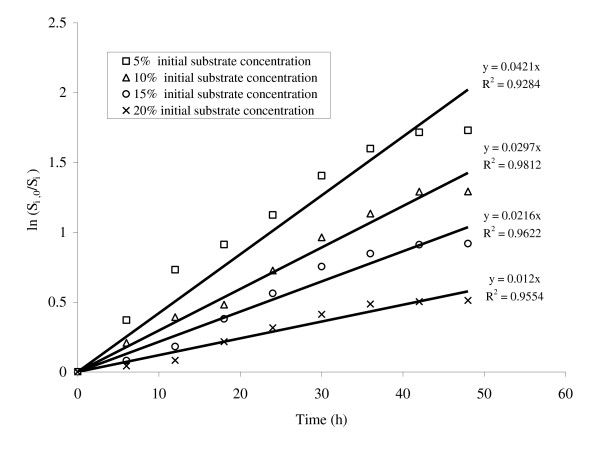
**Plot between substrate concentrations versus time for the enzymatic saccharification of delignified lignocellulosic biomass at different initial substrate consistencies**.

A series of batch experiments were performed using the initial substrate concentrations 5%, 10%, 15% and 20% (w/v). Then applying the kinetic model the rate constants of cellulose hydrolysis, *k_i _*(*i *= 1-4) was calculated in each case. It was observed that the rate constant for enzymatic hydrolysis decreases with an increase in the initial insoluble solid concentration (Figure 1). However, the *k_i _*values have shown good correlation with the initial substrate consistencies used for enzymatic hydrolysis with a regression coefficient R^2 ^of ~0.9.

**Figure 2:**
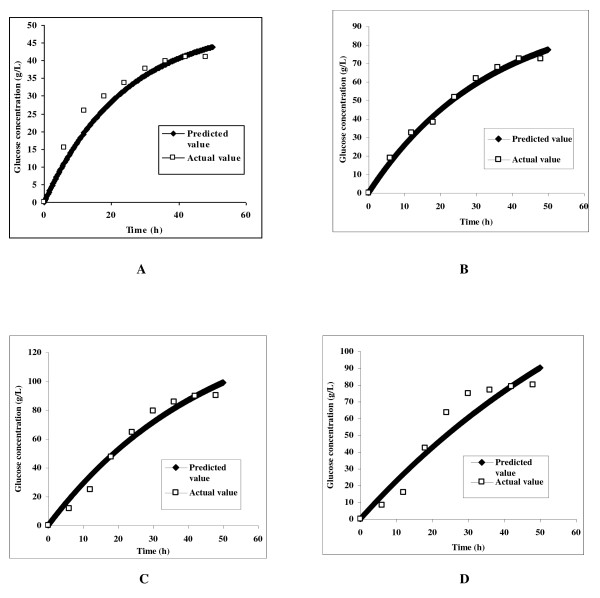
**Plots between the actual and the predicted values of glucose concentration released during the enzymatic hydrolysis of delignified lignocellulosic biomass at 5% (A), 10% (B), 15% (C) and 20% (D) initial substrate consistencies**.

The maximum rate constant (*k*_1 _= 0.0421 h^-1^) was obtained when the hydrolysis was carried out with 5% initial substrate concentration. The rate constants *k_i _*were then validated using the glucose concentration measurements obtained during the hydrolysis experiments. The root mean square error (RMSE) values between the predicted and the experimental values for enzymatic saccharification carried out at 5, 10, 15 and 20% initial substrate consistency were 0.997, 0.779, 1.843 and 1.995, respectively (Figure 2 a-d). The results also indicated that the maximum deviation of the experimental data from the model prediction was observed when the enzymatic saccharification was carried out at 20% initial substrate consistency. Moreover, the experimental values also depicted that the sugar concentration increased significantly only upto 15% substrate consistency and declined thereafter at 20% substrate level (Figure 2 a-d). The maximum sugar concentration obtained at each substrate concentration were 41.10 g/L (*S*_1,0 _= 5%), 72.47 g/L (*S*_2,0 _= 10%), 90.07 g/L (*S*_3,0 _= 15%) and 80.05 g/L (*S*_4,0 _= 20%) (see Figure 2 a-d).

### Kinetics of fed-batch enzymatic hydrolysis

**Figure 3:**
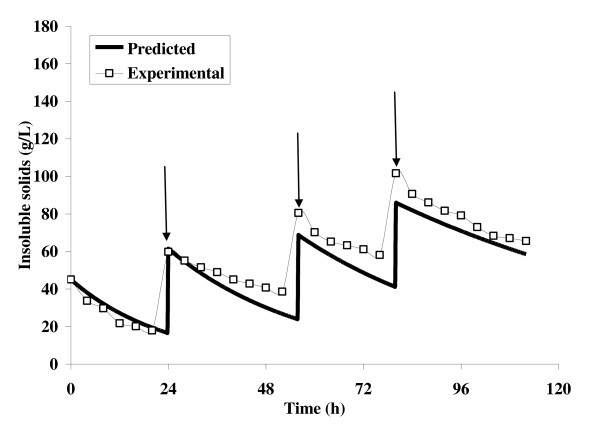
**Plot between the experimental versus predicted insoluble solids during the fed-batch enzymatic hydrolysis**.

The kinetic parameters determined from the batch experiments were used to simulate the hydrolysis profile during the fed-batch enzymatic hydrolysis. Fed-batch hydrolysis was performed employing discrete feeding policy. Insoluble solid substrate concentration (50 g) was added at 24, 56 and 80 h. The insoluble solid concentration was measured at 4 h intervals. A similar pattern of pulse responses were observed in both the experimental and predicted values for every instance of addition of 50 g feed to insoluble substrate (Figure 3). However, the final insoluble substrate concentrations for both the predicted and experimental values were 58.52 and 65.59 g/L, respectively (Figure 3).

### Comparison between batch and fed-batch enzymatic hydrolysis

**Figure 4:**
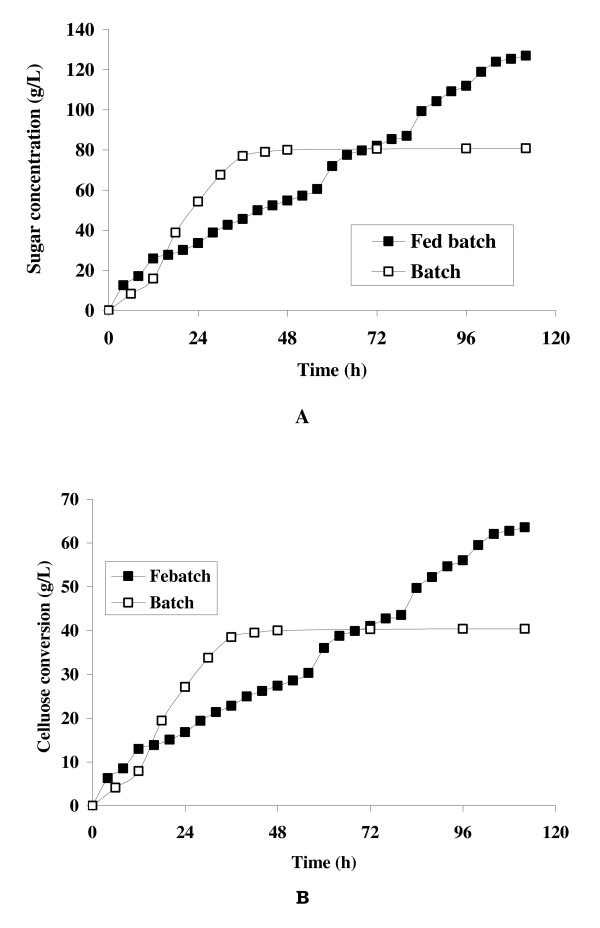
**Comparative profiling of batch and fed-batch enzymatic saccharification (A) glucose production, (B) cellulose conversion**.

A comparison between batch and fed-batch enzymatic hydrolysis was made to determine which had a higher cellulose conversion. The results showed that in fed-batch operation at *S_c _*= 20%, the remaining insoluble solids in the reaction slurry was 65.59 g/L. While in contrast, during the batch enzymatic hydrolysis at *S*_4,0 _= 20% (w/v), the concentration of remaining insoluble solid was 107.29 g/L. The time profiles of glucose concentration and cellulose conversion levels for both batch (*S*_4,0 _= 20%) and fed-batch (*S_c _*= 20%) were plotted in Figure 4a-b. The final sugar concentration for batch and fed-batch were 80.78 g/L and 127. 0 g/L (Figure 4a), while the cellulose conversion was 40.39% and 63.56%, respectively (Figure 4b). These results showed that intermittent addition of solids in a repeated fed-batch mode, resulted in better conversion compared to the addition of an equal amount once at the beginning of a batch.

### Simulation of kinetic model

**Figure 5:**
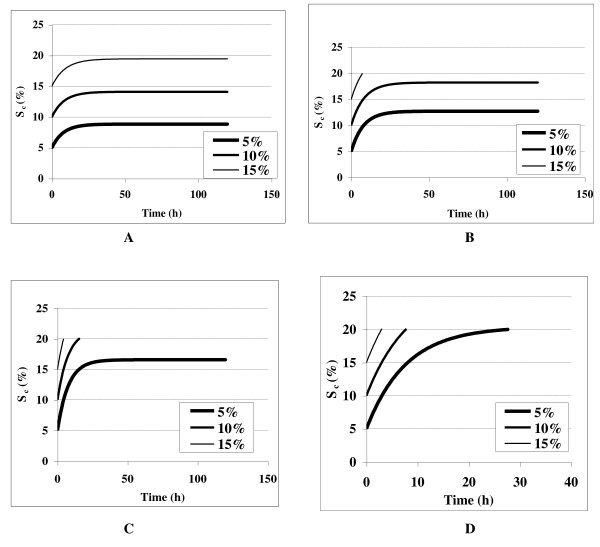
**Kinetic simulation profile for cumulative insoluble solids feeding at different initial substrate concentration (5-15%) at varied dilution rate of (A) 0.1 h^-1^, (B) 0.2 h^-1^, (C) 0.3 h^-1 ^and (D) 0.4 h^-1^**.

**Figure 6:**
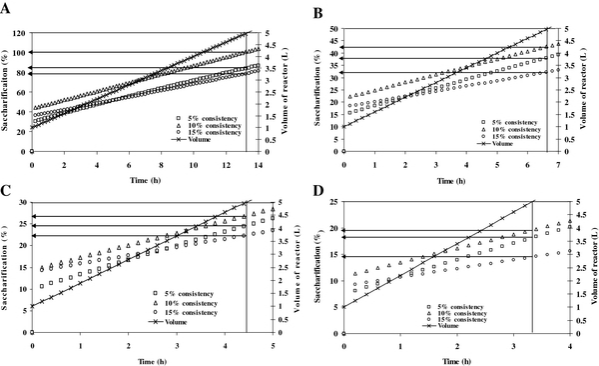
**Kinetic simulation profile for the volume of bioreactor at different initial substrate consistency (5-15%) using dilution rate of (A) 0.1 h^-1^, (B) 0.2 h^-1^, (C) 0.3 h^-1 ^and (D) 0.4 h^-1^**.

The kinetic parameters determined from the batch experiments were used to simulate different feeding policies. These simulations provide an insight about the operational protocol that may be implemented to obtain the best hydrolysis results. The simulation of discussed kinetic model under the fed-batch optimization approach has been shown in Figure 5 and 6. The Figure 5 depicted four different feeding policies developed from simulations with the target cumulative insoluble solids in the reactor as 20%. The simulation results showed that the cumulative insoluble solid concentration increases with time and saturates at different final values depending on the initial feed concentration at dilution rates of 0.1-0.4 h^-1 ^(Figure 5). The results indicated that long residence times are required to reach these higher solids levels, when solids were controlled at 5% or lower (Figure 5). While, the simulation results in Figure 6 indicated that higher insoluble solids levels in the feed resulted in both smaller reactor volumes and shorter residence times to achieve a given feeding objective. However, it has been predicted from the simulation results that the feed controlled at 10% initial solid levels resulted in maximum saccharification.

### Fermentation of enzymatic hydrolysate

**Figure 7:**
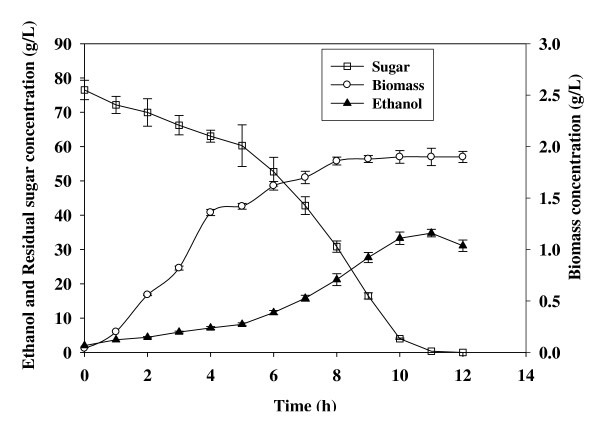
**Fermentation profile of batch enzymatic hydrolysate**.

**Figure 8:**
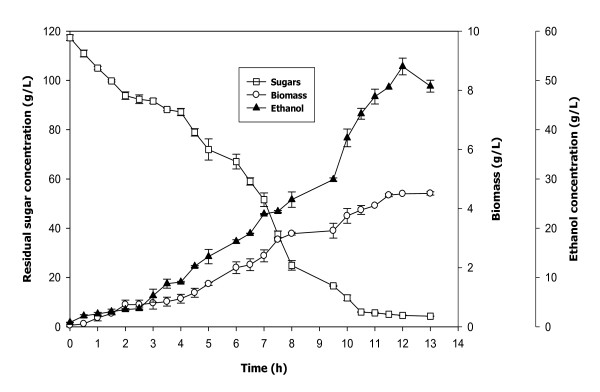
**Fermentation profile of fed-batch enzymatic hydrolysate**.

The fermentation profiles of batch (*S*_4,0 _= 20%) and fed-batch (*S_c _*= 20%) enzymatic hydrolysates containing 76.52 ± 2.82 and 117.35 ± 1.14 g/L initial sugars have been shown in Figure 7 and 8. The fermentation of batch enzymatic hydrolysate brought about the production of 34.78 ± 1.10 g/L ethanol with yield and productivity of 0.45 g/g and 3.16 g/L/h, respectively, after 11 h of incubation (Figure 7). Moreover, the biomass production during the fermentation of batch enzymatic hydrolysate increased till 8 h (1.86 ± 0.04 g/L) and then remained almost constant (Figure 7). While, the fed-batch enzymatic hydrolysate when fermented with *S. cerevisiae*, produced 52.83 ± 1.70 g/L ethanol and 4.50 ± 0.004 g/L biomass with an ethanol yield of 0.45 g/g and ethanol productivity of 4.40 g/L/h after 12 h of incubation (Figure 8).

## Discussion

The main aim of the present investigation was to achieve high ethanol concentration as the final ethanol concentration in the fermentation broth is critical to make a cost-effective ethanol production process. Since the ethanol concentration is directly proportional to the sugar concentration, hence high concentration sugar syrup is a prerequisite. In the present study, the process modeling consisting of mass balance and kinetic models were used to provide insights into the process performance and to optimize the process for enhanced enzymatic hydrolysis. During the batch saccharification at different consistencies, a regular decrease in the rate constant with increase in the substrate concentration was observed (Figure 1) and the reaction was assumed to be a first order reaction. This decrease in rate may be attributed to the product inhibition, improper heat and mass transfer and the thermal deactivation of enzymes [7,12]. The difference between the experimental values and those predicted through simulation for our batch experiments at 20% insoluble solid consistency may be attributed to the same reasons (Figure 2d).

To overcome this problem in batch operation, fed-batch enzymatic hydrolysis was implemented. This approach exploits the property of cellulose solubilization during the enzymatic hydrolysis to increase the solid loading to the reactor, which otherwise would be difficult to handle if the entire insoluble solid was added initially. Interestingly, considering the fact that there are two phases present in the slurry, in the present study, the cellulose conversion has been mentioned in terms of g/L of actual liquid present in the slurry, which was a major pit fall in the earlier report [7], who reported the conversion in terms of g/Kg of total slurry. The later was further amended by correcting the measurement of glucose in the liquid phase (which may represent only 80-90% of the total mass of the slurry) for the content of insoluble solids in order to accurately estimate conversion [15].

The present study demonstrated that fed-batch hydrolysis resulted in higher solid saccharification with high saccharification yield. The results in the (Figures 4a and 4b) depicted a final sugar concentration of 127 g/L with ~64% cellulose conversion, which was significantly higher than the cellulose conversion at batch operation (*S*_4,0 _= 20%). It is estimated that an increase in solid substrate consistency from 5 to 8% in simultaneous saccharification and fermentation process (SSF) reduced the process cost by 19% [16]. While according to report by National Renewable Energy Laboratory (NREL), Department of Energy (DOE), US, an increase in solid consistency from 20 to 30% can reduce the minimum ethanol selling price by $0.10/gallon ethanol [17]. Therefore, the high final sugar concentrations obtained in this work may lead to an economically competitive process.

Comparison of the accuracy of the model prediction validated that a well-designed fed-batch approach could be used to allow an STR reactor capable of handling pretreated *P. juliflora *at below than 10% insoluble solids to operate at cumulative initial insoluble solids as high as the set goal of 20% (Figure 5). Moreover such validation are also in accordance with the earlier reports of Hodge and coworkers [7], according to whom, using fed-batch strategy the STR, was able to achieve very high cumulative solid loading (*S*_4,0 _= 20%), thus improving its working capability. In addition, the model may also be used to determine a fed-batch feeding policy required to maintain proper mixing and temperature control necessary for high cumulative insoluble solids.

The fermentation of the enzymatic hydrolysate obtained from batch and fed-batch operation also indicates the significance of the study. The fermentation of enzymatic hydrolysate from fed-batch operation brought about approximately 50% and 40% increment in the ethanol concentration and the ethanol productivity, respectively. As there have been estimations that by doubling the ethanol concentration from 2.5 to 5%, the energy required to distill a fermentation broth to 93.5% ethanol using conventional distillation techniques can be reduced by 33% [9]. The enhanced ethanol concentration and productivity from fed-batch operation also made the process more industrially realistic.

## Conclusion

To produce higher concentration sugar syrup and subsequently the high ethanol concentration, fed-batch enzymatic saccharification was conducted with the pretreated *P. juliflora*. Through the fed-batch process, the cumulative solid loading (*S_c_*) up to 20% in a stirred tank reactor increased the sugar released by 56% compared to the batch process with an initial insoluble solid loading of 20%. This model used here provided additional insight into the effect of the operational conditions on productivity. This may be refined by including the degree of polymerization of substrate, accessible cellulose fraction, crystallinity of substrate and enzyme adsorption to distinguish the various causes of the decreasing rate of reaction.

## Methods

### Raw material and chemicals

*Prosopis juliflora *wood, collected from University of Delhi South Campus, New Delhi, India, was comminuted by a combination of chipping and milling to attain a particle size of 1-2 mm using a laboratory knife mill (Metrex Scientific Instrumentation, Delhi, India). The processed wood of *P. juliflora *was delignified with 4% sodium chlorite at 120 C for 30 minutes as described earlier [18].

Commercial cellulases and 3,5-di nitro salicylic acid (DNS) were purchased from Sigma (St. Louis, Missouri, U.S.A.). Ethanol was purchased from Merck (Darmstadt, Germany). Rest of the chemicals and media components of highest purity grade were purchased locally.

### Microorganism and culture conditions

The yeast *Saccharomyces cerevisiae *HAU procured from the culture collection of C.C.S. Haryana Agricultural University, Hisar, Haryana, India was maintained on agar slants containing (g/L): glucose, 30.0; yeast extract, 3.0; peptone, 5.0; agar, 20.0 at pH 6.0 ± 0.2 and temperature 30°C, as described earlier [1,8]. While the *S. cerevisiae *inoculum was grown for 24 h at 30°C in a culture medium containing (g/L): glucose, 30.0; yeast extract, 3.0; peptone, 5.0; (NH_4_)_2_HPO_4_, 0.25 at pH 6.0 ± 0.2 [1,19]. Cells were cultured to an absorbance of 0.6-0.8 at 600 nm.

### Enzymatic hydrolysis

#### Batch enzymatic hydrolysis

Enzymatic hydrolysis of pretreated substrate was carried out at different substrate consistency (5-20% w/v) in 0.05 M citrate phosphate buffer (pH 5.0) in a 3.0 L stirred tank bioreactor (Scigenics Pvt. Ltd, Chennai, India) fitted with Rushton impellors, heating jacket and heat exchangers for proper agitation and temperature control. Before enzyme loading, slurry was acclimatized by incubating at 50°C at 150 rpm for 2 h. Thereafter, an enzyme (lyophilized) dosage of 22 Filter paper cellulase activity (FPU)/g dry substrate (gds), 68 U β-glucosidase/gds was added to preincubated cellulose slurry, and reaction was continued for 48 h. One percent Tween 80 and 1 mM CuCl_2 _were also added to facilitate the enzymatic reaction. The samples were withdrawn at regular intervals, centrifuged at 10,000 rpm for 10 min and the supernatants were used for further analysis.

#### Fed-batch enzymatic hydrolysis

Fed-batch enzymatic saccharification of pretreated substrate was carried out in the same bioreactor with an initial substrate consistency of 5% (w/v) in the suspension. Before enzyme loading, the slurry was acclimatized by incubating at 50°C at 150 rpm for 2 h. Thereafter, an enzyme dosage of 22 FPU/gds and 68 U β-glucosidase/gds, 1% Tween 80 and 1 mM CuCl_2 _was added to preincubated cellulose slurry. The equal amount of initial substrate and half of the initial enzyme (lyophilized) was added to the enzymatic suspension thrice after 24, 56 and 80 h to get a final substrate concentration of 200 g/L. The samples were withdrawn at regular intervals, centrifuged at 10,000 rpm for 10 min and the supernatant was subjected to sugar estimation. After incubation, the hydrolysate was harvested, centrifuged to remove the un-hydrolyzed residues and the filtrate was used for fermentation studies.

### Fermentation of enzymatic hydrolysate

The fermentation studies of both the enzymatic hydrolysates from batch operation (*S*_4,0 _= 20%) and fed-batch operation (*S_c _*= 20%) were carried out. The batch and fed-batch enzymatic hydrolysates containing 37 g/L and 120 g/L sugars, respectively, supplemented with 3 g/L yeast extract and 0.25 g/L (NH_4_)_2_HPO_4_, were inoculated with 6% (v/v) *S. cerevisiae*. The fermentation was carried out at 30°C, 200 rpm and initial pH 6.0 ± 0.2. Aeration of 0.4 vvm was maintained throughout the study. The pH was adjusted with 2 N HCl and 2 N NaOH. The samples withdrawn were centrifuged at 10,000 rpm for 10 min at 4°C and the cell free supernatant was used for the determination of ethanol produced and sugar consumed.

### Kinetics and theoretical aspects of batch and Fed-batch enzymatic hydrolysis

Cellulose conversion is commonly used as a measure of the effectiveness of enzymatic hydrolysis of cellulose. The conversion efficiency (ξ) is described in terms of cellulose conversion to glucose (*G*) and the initial cellulose concentration (*S *_*i*,0_), given by

(1) $$\xi =\frac{G}{1.11}\left(\frac{1}{S_{i,0}}\right)$$

The insoluble solids level can be measured from the change in sugar concentrations relative to the initial cellulose concentration. This change in sugar levels can be related stoichiometrically to the amount of cellulose removed from the solid phase to estimate an insoluble solids level.

(2) $$S_{i}=S_{i,0}-\frac{G}{1.11}$$

The equations describing the dynamic changes in *S_i _*and *G *are

(3) $$\frac{dS_{i}}{dt}=-k_{i}S_{i}$$

where *k_i_, i *= 1 - 4, are the rate constants for different loadings, and

(4) $$\frac{dG}{dt}=1.11k_{i}S_{i,0}-k_{i}G$$

These equations can be solved analytically to obtain following relation

(5) $$G\left(t\right)=1.11S_{i,0}\left(1-\text{exp}\left(-k_{i}t\right)\right)$$

#### Mass balance equation for prediction of fed-batch capabilities

Mass balances were performed on the reaction system to evaluate the fed-batch procedure. A key assumption was that the insoluble solids (*S_i_*) are fed at a fixed flow rate (*F*). The final insoluble solid consistency obtained from the mass balance on insoluble solids at any time point is given by

(6) $$S=S_{F}+S_{i}-k_{i}S_{i}$$

Where *S *is the final insoluble solid concentration and *S_F _*is the concentration of solids fed. The cumulative insoluble solid (*S_c_*) is the sum of the total amount of insoluble solids present initially and the amount of substrate fed to the reactor. It would represent the level of solid that would be present if the entire solid were added initially and the reactor was operated in batch mode to enable comparison of fed-batch performance with the batch reactor performance on an equivalent basis.

For fed-batch operation, the model provides the rate expression for concentration of cellulose in the insoluble solid, glucose and cellulase enzyme. In addition to these variables, the dilution rate (*D*) was introduced to account for the changing mass and concentration due to a feed stream and is defined as the feed flow rate (*F*) per volume (*V*). The equations for fed-batch operations are as follows:

(7) $$\begin{array}{c} \frac{dS_{i}}{dt}=-k_{i}S_{i}+D\left(S_{F}-S_{i}\right)\\ \frac{dG}{dt}=1.11k_{i}S_{i}+D\left(G_{F}-G\right)\\ \frac{dE}{dt}=D\left(E_{F}-E\right)\\ \frac{dV}{dt}=F \end{array}$$

#### Fed-batch saccharification model simulation

Using the above kinetic model, a feeding policy was developed based upon controlling the insoluble solids below a defined critical value during the saccharification reaction. This is possible by feeding a stream of pretreated substrate at a rate that approximately matches the rate of saccharification. Using the kinetic model equations, the rate of change of insoluble solids can be determined with the set of initial operating conditions and the insoluble solids at any given time point [*S_i_*(t)] will be,

(8) $$S_{i}\left(t\right)=\frac{S_{F}D}{\left(k_{i}+D\right)}\left(1-\text{exp}\left(-k_{i}t\right)\right)$$

Using this algorithm, fed-batch feeding policies were developed by generating a set of feeding curves over various reactor solids concentration and initial conditions generated to determine within the theoretical physical limitation of the system and the potential for using a fed-batch approach.

### Analytical methods

The cellulase activities were determined following International Union of Pure and Applied Chemistry (IUPAC) methods [20]. The hydrolysates were analysed using high performance liquid chromatography (HPLC) (Waters, USA) for the presence of carbohydrates. Carbohydrate-ZX (Agilent Technologies, USA) column (300.0 × 7.8 mm) was used with Milli-Q water as an eluent with flow rate of 1.0 mL/min keeping oven temperature at 30 C with RID detector. Ethanol was estimated by gas chromatography (GC) (Perkin Elmer, Clarus 500) with an elite-wax (cross bond-polyethylene glycol) column (30.0 m × 0.25 mm), at oven temperature 90°C and flame ionization detector (FID) at 200°C. Nitrogen with a flow rate of 0.5 mL min^-1 ^was used as carrier gas.

## Competing interests

The authors declare that they have no competing interests.

## Authors' contributions

RG carried out the experimental work, analyzed the results and drafted the manuscript. SK helped in preparation of kinetic data and in technical checking of manuscript paper. JG designed the kinetic model and discussed the analysis of their results RCK coordinated the overall study and helped to analyze the results and finalize the paper. All authors read and approved the final manuscript.
